# FORGIVENESS AS A RESOURCE FOR COUNTERING FEELINGS OF LONELINESS AMONG WOMEN AGING IN CORRECTIONAL CUSTODY

**DOI:** 10.1093/geroni/igae098.2550

**Published:** 2024-12-31

**Authors:** Anya Thompson, Alex Bishop, Kevin Randall

**Affiliations:** Oklahoma State University, Stillwater, Oklahoma, United States; Oklahoma State University, Stillwater, Oklahoma, United States; Sam Houston State University, Huntsville, Texas, United States

## Abstract

Analyzing cross-sectional data from 232 women in custody ages 40-years and older (M = 49.06; SD = 7.22), we examined a conceptual path analytic model based on the developmental adaptation model consisting of distal and proximal influences on Loneliness, controlling for age, race, education, marital status, and crime type. The tested model linked participant recall of adverse childhood experiences (ACES) to loneliness (LONE) through religious doubt (RD) and three assessments of dispositional forgiveness, Self, Others, and Situation. The model fit the data well, X2 = 2.09 (3 df); p =.55; RMSEA = 0.00; CFI = 1.00; and SRMR =.008. Standardized results revealed that RD was related to ACES (.13, one-tailed p =.028). In addition, significant paths were found linking all three disposition assessments of forgiveness to RD: Self, β = -.42, p <.001; Others, β = -.32, p <.001; and Situation, β = -.47, p <.001. However, only Self was significantly associated with LONE: β = -.19, p =.017; however, there was a significant total indirect effect on LONE from ACES through RD and the three measures of forgiveness;.019, one-tailed p =.045. The model explained 22% of the variance in LONE. Results have implications for how clinical counselors, social workers, cases managers, and pastoral care ministers administer rehabilitative programming. Discussion will highlight forgiveness as a rehabilitative resource for addressing childhood trauma and religious doubt, as well as reducing feelings of loneliness reported by middle aged women in correctional custody.

